# Isolation of endophytic fungi from *Cotoneaster multiflorus* and screening of drought-tolerant fungi and evaluation of their growth-promoting effects

**DOI:** 10.3389/fmicb.2023.1267404

**Published:** 2023-11-02

**Authors:** Zeng-Wei Lü, Hao-Yang Liu, Chen-Lu Wang, Xue Chen, Yi-Xiao Huang, Miao-Miao Zhang, Qiu-Liang Huang, Guo-Fang Zhang

**Affiliations:** College of Forestry, Fujian Agriculture and Forestry University, Fuzhou, China

**Keywords:** *Cotoneaster multiflorus*, endophytic fungi, PEG-6000, drought tolerance, growth promotion

## Abstract

In the context of climate change and human factors, the drought problem is a particularly serious one, and environmental pollution caused by the abuse of chemical fertilizers and pesticides is increasingly serious. Endophytic fungi can be used as a protection option, which is ecologically friendly, to alleviate abiotic stresses on plants, promote plant growth, and promote the sustainable development of agriculture and forestry. Therefore, it is of great significance to screen and isolate endophytic fungi that are beneficial to crops from plants in special habitats. In this study, endophytic fungi were isolated from *Cotoneaster multiflorus*, and drought-tolerant endophytic fungi were screened by simulating drought stress with different concentrations of PEG-6000, and the growth-promoting effects of these drought-tolerant strains were evaluated. A total of 113 strains of endophytic fungi were isolated and purified from different tissues of *C. multiflorus*. After simulated drought stress, 25 endophytic fungi showed strong drought tolerance. After ITS sequence identification, they belonged to 7 genera and 12 species, including *Aspergillus, Fusarium, Colletotrichum, Penicillium, Diaporthe, Geotrichum*, and *Metarhizium*. According to the identification and drought stress results, 12 strains of endophytic fungi with better drought tolerance were selected to study their abilities of dissolving inorganic phosphorus and potassium feldspar powder and producing indole-3-acetic acid (IAA). It was found that the amount of dissolved phosphorus in 7 strains of endophytic fungi was significantly higher than that of CK, and the content of soluble phosphorus was 101.98–414.51 μg. ml^−1^; 6 endophytic fungi had significantly higher potassium solubilization than CK, and the content of water-soluble potassium ranged from 19.17 to 30.94 mg·l^−1^; 6 strains have the ability to produce IAA, and the yield of IAA ranged between 0.04 and 0.42 mg. ml^−1^. This study for the first time identified the existence of endophytic fungi with drought tolerance and growth-promoting function in *C. multiflorus*, which could provide new direction for plant drought tolerance and growth promotion fungi strain resources. It also provides a theoretical basis for the subsequent application of endophytic fungi of *C. multiflorus* in agricultural and forestry production to improve plant tolerance.

## 1. Introduction

Endophytic fungi are a group of important microbial resources that grow in healthy plant tissues and achieve mutual benefit and symbiosis with their hosts (Azevedo et al., 2000). Relevant studies have found that endophytic fungi are associated with the growth, development, and stress resistance of plants during the process of co-evolution with plants (Hendry et al., 2009; Zhang et al., 2019). It has been discovered that each plant contains approximately 4–5 endophytic fungi, and the total number of endophytic fungi can exceed one million based on the calculation of known plant species on Earth, which is approximately 250,000 (Jia et al., 2016).

Due to the “selection” of the host, the distribution of endophytic fungi in different tissues of the host is different, and the effects on different tissues are also different (Edwards et al., 2015). According to relevant studies, at present, endophytic fungi in plants mostly belong to the phylum of dinucleate fungi, and the subclass of Ascomycotina includes Pyrenomycetes, Discomycetes, and Loculoascomycetes, as well as a variety of fungi without morphology (Carroll, 1988), their hosts include algae, coniferous trees, shrubs, and herbs. In addition, endophytic fungi have established a close relationship with host plants through long-term symbiosis. In addition to promote the growth and development of host plants through a variety of mechanisms (Nazia et al., 2021, 2022), endophytic fungi can also produce secondary metabolites with the same or similar physiological activities as host plants (Mayerhofer et al., 2013). It has good research and development potential in many research fields such as agriculture and forestry (Narware et al., 2023). Endophytic fungi with bacteriostatic, growth-promoting, and anti-stress activity have been isolated from *Glycine max* (L.) Merr. (Petrini, 1991), *Vaccinium uliginosum* L. (Qu et al., 2022), *Commelina communis* L. (Liu et al., 2023), *Vernicia montana* Lour. (Zhang et al., 2016), *Pogostemon cablin* (Blanco) Benth. (Wang et al., 2017), and other plants. In addition, endophytic fungi can also decompose a variety of organic matter such as sugar and tannin and participate in the formation and decomposition of humus, ammonification, and nitrification. Therefore, they play an important role in promoting the growth of host plants and have a positive impact on the growth indices of host plants (Cao, 2018; Mondal et al., 2020; Kashyap et al., 2021).

With global warming and the interference of human activities, drought has seriously affected the growth, development, quality, and yield of plants (Xu et al., 2020). Related studies have found that improving plant stress adaptability by exploring endophytic microorganisms is a new direction for plant drought resistance research. In recent years, it has been reported that drought-tolerant endophytic fungi can be isolated from plants. For example, Ou et al. (2023) screened out *Trichoderma* GS1, *Pseudopteris* GRs12, *Penicillium* GR19, and *Trichoderma* GR21 by applying periodic drought stress to *Morus alba* L. The mixed suspension can improve the drought tolerance of mulberry by inducing the production of catalase, soluble sugar, and chlorophyll. A065, a dark septonic endophytic fungus selected from *Pinus sylvestris* var. *mongolica* Litv., has significant drought tolerance, and its colony diameter and biomass at a PEG concentration of 30% are significantly higher than those of other strains (Shao et al., 2022).

Morphological and genetic identification plays a crucial role in microbiological research. Morphological identification relies on the observation and description of external features, while genetic identification utilizes molecular biology techniques to analyze genetic information, such as DNA sequences (Gardes and Bruns, 2010). The Internal Transcribed Spacer (ITS) genes are commonly used genetic markers due to their high variability and stability, making them suitable for species identification and phylogenetic studies. By using ITS sequences for genetic identification, the taxonomic identity of each microbial strain can be determined (Schoch et al., 2015). Currently, ITS sequencing is widely applied in fungal classification, species identification, and phylogenetic research.

*Cotoneaster multiflorus* Bunge. has been widely used in urban landscaping and forestation of barren mountains with its characteristics of strong adaptability and resistance to coldness, drought, and barrenness. At the same time, its branches, leaves, and fruits can be used in medicines to treat joint muscle rheumatism, gingival bleeding, and other diseases (Jia and Xu, 2014). On the basis of the secondary metabolites, propagation, and breeding of plants, a large number of effective research studies have been conducted on *C. multiflorus*. However, endophytic fungi colonization and germplasm resources on *C. multiflorus* are still insufficient. It is also unclear whether there are drought tolerant and growth promoting endophytic fungi in *C. multiflorus*. In this study, we isolated endophytic fungi from the roots, stems, and leaves of *C. multiflorus*, screened out the drought-tolerant endophytic fungi by PEG-6000 under simulated drought conditions, and evaluated the growth promotion ability of these drought-tolerant strains. The comprehensive analysis of probiotics on *C. multiflorus* will provide a theoretical basis for the rational development and utilization of drought-tolerant fungi and new strain resources for drought resistance and growth promotion.

## 2. Materials and methods

### 2.1. Materials and reagents

The samples were obtained from Liupanshan National Nature Reserve (near Longtan Forest Farm), Ningxia Hui Autonomous Region (106°09–106°30 'E, 35°15′ −35°41 'N), collected in July 2022. Ten 5 × 5 m quadrats were set in the growing area of *C. multiflorus*, and the distance between each quadrat was more than 100 m. A total of 10 healthy wild *C. multiflorus* plants aged 3–5 years were randomly selected from each quadrat. The whole plant was dug out while collecting and brought back to the laboratory with soil for subsequent experiments.

The test medium includes:

PDA medium: PDA (Potato Dextrose Agar) 40:1 g, distilled water 1,000 ml, and streptomycin 0.03 g.

Inorganic phosphorus (NPA) liquid medium: MnSO_4_·H_2_O 0.03 g, Glucose 10 g, (NH_4_)_2_SO_4_ 0.5 g, NaCl 0.3 g, KCl 0.3 g, FeSO_4_·7H_2_O 0.03 g, Ca_3_(PO_4_)_2_ 5.0 g, H_2_O 1,000 ml, pH 7.2, and MgSO_4_·7H_2_O 0.3 g, and solid medium was supplemented with 20 g of agar.

Dissolved potassium medium: sucrose 10.0 g, potassium feldspar powder 1 g, yeast extract 0.5 g, MgSO4^.^7H_2_O 0.5 g, (NH_4_)_2_SO_4_ 1.0 g, Na_2_HPO_4_ 2.0 g, CaCO_3_ 1.0 g, and distilled water 1,000 ml, and solid medium was supplemented with 20 g of agar.

### 2.2. Methods

#### 2.2.1. Isolation and purification of endophytic fungi

The plate dilution method was used to isolate and purify endophytic fungi from different tissues of *C. multiflorus*. Germ-free roots, stems, and leaves of 10 wild *C. multiflorus* plants were collected and washed with sterile water. After drying with sterile filter paper, 1 g of each tissue part was weighed. The leaves were soaked in 75% ethanol for 30 s, while the stems and roots were soaked for 60 s. They were, then, washed with sterile water five times and dried. Subsequently, the tissue samples were immersed in 3–10% sodium hypochlorite solution for 3–5 min, followed by washing with sterile water five times and drying with a sterile filter paper (Zhou et al., 2021; Baozhen et al., 2023). Then, 200 μl sterile water of the last rinse was pipetted and spread on a PDA medium plate to verify the disinfection effect. After surface disinfection, the material was placed on a sterile ultra-clean work table and fully ground with sterile mortar by adding sterile water. The tissue fluid was obtained by grinding and diluting using a gradient of 10^−2^, 10^−3^, 10^−4^, and 10^−5^. Next, 0.1 ml of each dilution was evenly spread on a PDA medium, with three replicates for each sample. Finally, they were cultured in an incubator at 28°C. After 3–5 days of culture, mycelium grew on the PDA medium, different mycelium tips were picked out by the tip of the inoculation needle and planted in the center of the PDA medium for purification. As for 3–7 days of culture in the dark environment at 28°C, the colonies with the most similar morphology were classified into the same type according to the color and morphology of the colonies and then numbered uniformly. G represents the root, J represents the stem, and Y represents the leaf. Numbered strains were placed on a slant at 4°C and stored until use.

#### 2.2.2. Selection of drought-tolerant strains of *C. multiflorus*

##### 2.2.2.1. Concentration settings

The purified endophytic fungi obtained from the aforementioned isolation were assessed for drought tolerance. To simulate drought stress, different concentrations of polyethylene glycol (PEG-6000) were added to the PDA medium. Following the experimental design of Botong et al. (2022), 0, 50, 100, 150, and 200 g of PEG-6000 were weighed and dissolved in sterile water, with the final volume fixed at 1,000 ml.

Five different levels of PEG-6000 osmotic potential were set as follows: PDB liquid culture medium with osmotic potential of 0 MPa was used as control (CK); PDB liquid culture medium with 5% PEG-6000 was used at the permeability action potential of −0.1 MPa; PDB liquid culture medium with 10% PEG-6000 was used at the permeability action potential of −0.2 MPa; PDB liquid culture medium with 15% PEG-6000 was used at the permeability action potential of −0.4 MPa; PDB liquid culture medium with 20% PEG-6000 was used at the osmotic potential of −0.6 MPa.

The preparation of standard endophytic fungi suspension is as follows: The endophytic fungi growing on the inclined plane of PDA were inoculated into a solid PDA medium for activation culture. After activation, the actively growing endophytic fungi were selected and inoculated into a sterilized PDB liquid medium. The cultures were, then, placed in a shaking incubator at a constant temperature of 28°C at 160 r.min^−1^ for 3 days. After 3 days, the optical density (OD600) value of each endophytic fungi suspension was measured. Each endophytic fungi solution was diluted with sterile water to obtain an endophytic fungi suspension with the same optical density value as the endophytic fungi solution with the lower OD600 value (the absorbance of endophytic fungi suspension at 600 nm wavelength, OD_600_). This resulting suspension served as the standard endophytic fungi suspension.

##### 2.2.2.2. Determination of strain drought tolerance

According to the method described by Li et al. (2021), the experiment was conducted, using PEG-6000 to simulate drought stress experiments on endophytic fungi. The specific procedure was as follows: 0.1 ml of the previously prepared standard endophytic fungal suspension was absorbed and added to a PDB liquid medium containing different concentrations of PEG-6000. The cultures were, then, placed in a shaking incubator at a constant temperature of 28°C at 160 r·min^−1^. After 7 days, the endophytic fungal suspension was mixed and sampled to determine the OD600 value. The growth and reproduction status of the endophytic fungi were evaluated based on the OD value, allowing for the analysis of their drought tolerance.

#### 2.2.3. Molecular identification of drought-tolerant endophytic fungi

##### 2.2.3.1. Selection of endophytic fungal strains

The endophytic fungi that exhibited drought tolerance after undergoing PEG-6000 simulated drought stress were selected as the materials for further study. These drought-tolerant strains were, then, propagated extensively for subsequent experiments.

##### 2.2.3.2. Genomic DNA extraction of endophytic fungi

Endophytic fungal DNA was extracted and purified according to the kit instructions provided by Shanghai Paisenno Biotechnology Co., Ltd.

##### 2.2.3.3. PCR amplification of endophytic fungal genomes and sequencing

###### 2.2.3.3.1. Reaction system

The primers used are shown in Table 1. The total volume of 50.0 μl reaction solution included 1.0 μl genomic DNA (20 ng.μL^−1^), 5.0 μl 10 × buffer (containing 2.5 mM Mg^2+^), 1.0 μl Taq polymerase (5 u.μl^−1^), 1.0 μl dNTP (10 mM), and 1.0 μl DNTP (10 mM), 1.5 μl ITS1/ITS-F primer (10 uM), 1.5 μl TS4/ITS-R primer (10 μM), and 39.0 μl ddH_2_O.

**Table 1:** Primer design (Seliger et al., 1990).

**Primer name**	**Sequence**
ITS1	TCCGTAGGTGAACCTGCGG
ITS4	TCCTCCGCTTATTGATATGC
ITS-F	CCGTGTTTCAAGACGGG
ITS-R	CTTGGTCATTTAGAGGAAGTAA

###### 2.2.3.3.2. PCR amplification conditions

Predenaturation at 95°C for 5 min; denaturation at 95°C for 30 s; annealing at 58°C for 30 s; and extension at 72°C for 1 min. The final extension was performed for 7 min at 72°C for 35 cycles. After completion of the reaction, 3 μl of the PCR product was subjected to 1% agarose gel electrophoresis. To confirm whether the PCR amplification fragment is qualified. PCR products were recovered with the AxyPrep DNA gel recovery kit. Sequencing of the PCR products was performed by Shanghai paseno Company Ltd (Shanghai, China).

##### 2.2.3.4. ITS sequence analysis of endophytic fungi

The sequences of endophytic fungi were compared with data available in NCBI using BLAST search to estimate the phylogenetic relationships. The resulting sequences were aligned using ClustalX software, with gaps considered as missing data. The phylogenetic tree was constructed using Mega software version 3, employing the neighbor joining method and Kimura two-parameter distance calculation.

#### 2.2.4. Selection of drought-tolerant strains with phosphate-solubilizing ability

##### 2.2.4.1. Phosphorus solubilizing ability of preliminary screened (qualitative) strains

The drought-tolerant fungi screened above were initially screened for phosphorus solubilization ability. The selected drought-tolerant strains were cultured, and after 3–5 days, an activated strain was picked using a needle and inoculated into an inorganic phosphorus (NPA) medium. Each dish had four inoculation points with three replicates, and the strains were incubated at 28°C for 3–4 days. The colony diameter (d) and phosphorus solubilizing circle diameter (D) were measured, and the ratio of the phosphorus solubilizing circle diameter to the colony diameter (D/d) was used to qualitatively assess the strain's phosphorus solubilizing ability. A larger decomposition circle and a higher value of (D/d) indicated a stronger phosphorus solubilizing ability (Marra et al., 2011; Liu et al., 2015).

##### 2.2.4.2. Re-screening (quantitative) of strains with phosphorus solubilizing capacity

The soluble phosphorus content of the fermentation broth was determined using the molybdenum antimony colorimetric method. The specific operational steps are as follows (Liu et al., 2014):

Preparation of phosphorus standard solution: Bake KH_2_PO_4_ in an oven at 105°C for 5 h in advance. After cooling, weigh 0.22 g and dissolve it in 400 ml of water. Then, add 5 ml of concentrated sulfuric acid (to be added while stirring). Using a pipettor, draw 5 ml of the prepared solution and transfer it to a volumetric flask. Shake well and adjust the volume to 100 ml.

Molybdenum antimony resistance base solution: Dissolve 1 g of ammonium molybdate in 45 ml of deionized water. Then, add 15.3 ml of H_2_SO_4_. Next, add 10 ml of a 0.5% antimony potassium tartrate solution. Finally, add deionized water to make the total volume 100 ml.

Molybdenum antimony anti-chromogenic solution: Take 100 ml of the molybdenum antimony resistance base solution and add 1.5 g of ascorbic acid (prepared when needed).

Determination of standard curve (Na, 2020): Take the phosphorus standard solution (0, 1, 2, 4, 6, 8, and 10 ml) prepared above into a 50-ml volumetric flask. Add an appropriate amount of distilled water into the volumetric flask and then add 5 ml of the molybdenum antimony anti-reagent. Finally, adjust the volume to 50 ml with distilled water. After shaking and allowing it to stand, 0, 0.1, 0.2, 0.4, 0.6, 0.8, and 1.0 μg/ml phosphorus standard series solutions is obtained. Place them at room temperature for 5 h and use the blank phosphorus standard solution as the control. Use an ultraviolet spectrophotometer at a wavelength of 700 nm to determine the absorbance value and draw the standard curve. The soluble P content in the liquid medium can be calculated using the standard curve.

Determination of solution configuration: Five endophytic fungal blocks with a diameter of 6 mm were intercepted by a hole punch and were inoculated into a 50 ml inorganic phosphorus liquid (NPA) medium. Each treatment group was set up three times and incubated in a shaker at a constant temperature of 28°C at 150 r^.^min^−1^. After 10 days, the culture medium was centrifuged at 10,000 r^.^min^−1^ for 15 min, and 5 ml of the centrifuged supernatant was put into a 50 ml volumetric flask. Then, two drops of 2, 4-dinitrophenol indicator were added, and 4 mol^.^L^−1^ NaOH solution was added drop-by-drop to the conical flask until the liquid turned slightly yellow. Then, 1 mol^.^L^−1^ H_2_SO_4_ solution was added until the yellow color faded, and 5 ml molybdenum antimony anti-color agent was added. After rapidly shaking, distilled water was added to 50 ml of mark line position, and the OD value at 700 nm was measured by a UV spectrophotometer after 2 h. The OD value measured by the strain was substituted into the phosphorus standard curve to obtain the phosphorus mass concentration, and then, after shaking the flask culture, the effective phosphorus content produced by the strain was calculated according to the calculation formula. The effective phosphorus content was calculated as follows:


(1) $$\begin{array}{l} AP=\frac{\left(P\times V\times T_{s}\right)}{V_{0}}, \end{array}$$

where *P* is the mass concentration of phosphorus (μg^.^ml^−1^) checked in the standard curve; V is the constant volume (ml), 50 ml in this study; *Ts* is divided into multiple of sampling; *V*_0_ is the volume of fermentation broth (ml), which is 3 ml in this study; and AP refers to the available phosphorus content (μg^.^ml^−1^).

#### 2.2.5. Selection of drought-tolerant strains with potassium solubilizing ability

##### 2.2.5.1. Preliminary screening of endophytic fungi with potassium solubilizing ability (qualitative)

The endophytic fungi with drought tolerance were cultured on PDA Petri dishes. After 3–5 days, one needle was used to pick the activated strain and inoculate into the potassium solution medium. Each dish had 4 inoculation points and 3 replicates, and the strain was incubated at 28°C for 3–4 days. The diameter of the colony (d) and the diameter of the potassium solubilizing ring (D) were measured, and the ratio of the diameter of the potassium solubilizing ring to the diameter of the colony (D/d) was used to qualitatively judge whether the strain had the ability of solubilizing potassium. The larger the decomposition ring, the larger the value of (D/d), indicating the stronger ability of solubilizing potassium, otherwise weaker.

##### 2.2.5.2. Re-screening of endophytic fungi with potassium solubilizing capacity (quantitative)

Five endophytic fungal blocks with a diameter of 6 mm were intercepted by a borehole and inoculated into 50 mL potassium solubilizing fungal liquid medium, while the potassium solubilizing medium without endophytic fungi was used as blank control. Each treatment was repeated three times. After 10 days of shaking at 28°C and 150 r^.^min^−1^, the culture medium of potassium solubilizing endophytic fungi was centrifuged at 4°C and 6,000 r·min^−1^ for 10 min. Then, 0.5 ml of the supernatant was boiled with 5 ml concentrated sulfuric acid and 2 ml 30% hydrogen peroxide solution. In total, 30% hydrogen peroxide solution was added repeatedly several times until the viscous substance was completely digested, and the soluble potassium content was determined by flame spectrophotometer after the volume was fixed to 50 ml (Jun et al., 2022).

#### 2.2.6. Screening of drought-tolerant strains with IAA-producing capacity

##### 2.2.6.1. Initial screening for secretion of indole-3-acetic acid (qualitative)

###### 2.2.6.1.1. Preparation of Salkowski colorimetric solution

Take 12 g of FeCl_3_, dissolve it in 500 ml of distilled water, then slowly add 430 ml of 98% H_2_SO_4_ (add while stirring), cool and set the volume to 1 L, and configure as a standard colorimetric solution (Glickmann and Dessaux, 1995).

###### 2.2.6.1.2. Culture of endophytic fungi

Five endophytic fungal blocks with a diameter of 6 mm were collected using a borehole and inoculated into 50 ml of PDB liquid culture medium. The cultures were maintained at 28°C at 180 r·min^−1^ for 5–7 days. Afterward, the endophytic fungi suspension was obtained, and it was centrifuged at 12,000 r·min^−1^ for 10 min. Next, 2 ml of the supernatant was taken and mixed with an equal volume of Salkowski colorimetric solution. In parallel, a mixture of the same volume of uninoculated PDB liquid culture medium and Salkowski colorimetric solution was prepared as a blank control. Both samples were, then, placed in a dark environment at room temperature for 30 min. If the color remains unchanged, it indicates that the endophytic fungi do not produce indole-3-acetic acid (IAA). On the other hand, if the color turns pink, it indicates that the endophytic fungi are capable of producing IAA.

##### 2.2.6.2. Re-screening of indole-3-acetic acid-secreting endophytic fungi (quantification)

The standard solution was prepared by taking distilled water and 10 mg of IAA standard sample and diluting it to 100 ml (concentration of 0.1 mg^.^l^−1^). Then, 0, 2.5, 5, 7.5, 10, and 12.5 ml of the above standard solution were taken in 25 ml volumetric flasks, resulting in concentrations of 0, 10, 20, 30, 40, and 50 mg.ml^−1^, respectively. The IAA standard solutions were mixed with Salkowski chromogenic agent at a volume ratio of 1:1 and placed at room temperature, protected from light for 30 min. The absorbance of each concentration at OD_530_ was measured using an ultraviolet spectrophotometer, and the IAA standard curve was plotted with each concentration of the IAA standard as the x-axis and the corresponding absorbance as the y-axis.

###### 2.2.6.2.1. IAA content determination

Five 6 mm diameter endophytic fungi cakes were inoculated into 50 ml of PDB liquid culture medium by intercepting the newly screened IAA-producing strains with a hole punch and cultured in a shaker at 28°C. After fermentation for 72 h, the absorbance value of OD_530_ after the reaction of 1 ml of fermentation supernatant with Salkowski color agent was determined by pipetting the endophytic fungi suspension at 12,000 r·min^−1^, and then, the corresponding IAA content was calculated according to the IAA standard curve.

#### 2.2.7. Data statistics and analysis

Excel 2016 software was used to calculate the mean and standard error, and Duncan's new complex range method was carried out by SPSS 27.0 software to test the significance of differences (*P* < 0.05).

## 3. Results

### 3.1. Isolation of culturable endophytic fungi from different parts of *C. multiflorus*

In this experiment, the diluted plate method was used to isolate and culture endophytic fungi from the roots, stems, and leaves of *C. multiflorus* collected from Liupanshan National Nature Reserve. In total, 200 μl sterile water was applied to the PDA medium plates for culture. No fungal growth was observed, indicating thorough disinfection of the tissue samples and confirming that the isolated and purified endophytic fungi originated from the tissues. After repeated isolation and purification, 113 strains of endophytic fungi were obtained from *C. multiflorus*. The distribution of these strains is presented in Table 2, with 40 strains isolated from the roots (35.40% of the total), 45 strains from the stems (39.82% of the total), and 28 strains from the leaves (24.78% of the total).

**Table 2:** Isolation and purification results of endophytic fungi from different tissues of *C. multiflorus*.

**Plant tissue**	**Number of isolates obtained**
Root	40
Stem	45
Leaf	28
Total	113

**Figure 1:**
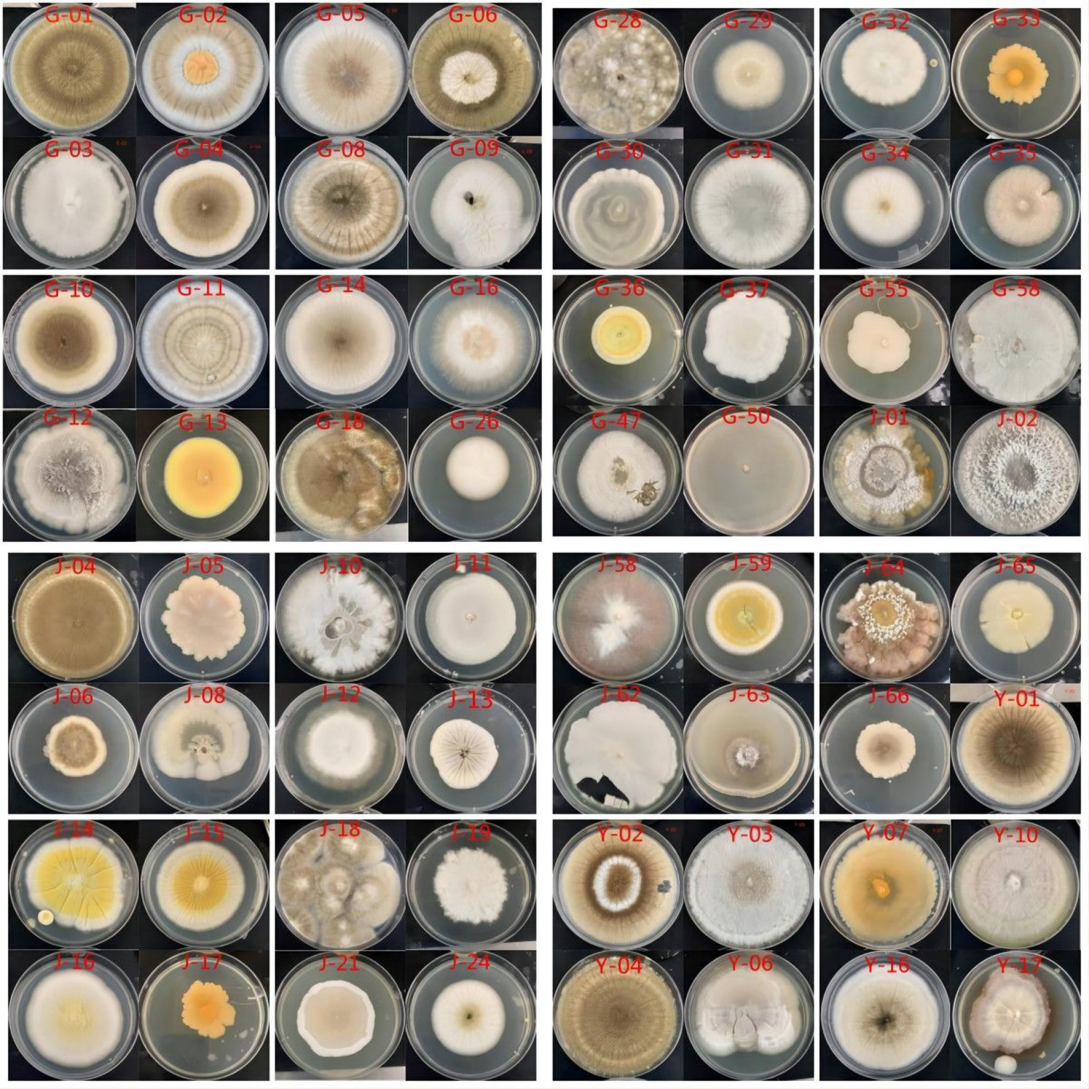
The colony morphology of endophytic fungi in different tissues of *C. multiflorus*.

The results of the isolation and purification of culturable endophytic fungi from *C. multiflorus* are illustrated in Figure 1.

### 3.2. Screening and identification results of drought-tolerant strains of *C. multiflorus*

#### 3.2.1. Screening results of drought-tolerant strains of *C. multiflorus*

The drought tolerance of endophytic fungi was assessed based on their ability to proliferate under different concentrations of simulated drought stress using PEG-6000. Table 3 shows the drought-tolerant strains that were able to proliferate at various concentrations of PEG-6000-induced drought stress. Among these strains, 13 were isolated from roots, 7 from stems, and 5 from leaves, accounting for 10.83, 5.83, and 1.67% of the total isolated drought-tolerant strains, respectively.

**Table 3:** Absorbance value (OD_600_) of endophytic fungi in different tissues of *C. multiflorus* under simulated drought stress.

**Strains**	**OD600 absorbance at different osmotic potentials (MPa)**									**Turbidity relative value/%**			
	**0**	−**0.1**	**Decrease/%**	−**0.2**	**Decrease/%**	−**0.4**	**Decrease/%**	−**0.6**	**Decrease/%**	−**0.1**	−**0.2**	−**0.4**	−**0.6**
G01	0.42	0.48	**–**14.29	0.51	**–**21.43	0.32	23.81	0.23	45.24	114.29	121.43	76.19	54.76
G02	0.31	0.36	**–**16.13	0.43	**–**38.71	0.28	9.68	0.19	38.71	116.13	138.71	90.32	61.29
G05	0.68	0.72	**–**5.88	0.65	4.41	0.52	23.53	0.36	47.06	105.88	95.59	76.47	52.94
G06	0.73	0.98	**–**34.25	0.46	36.99	0.37	49.32	0.25	65.75	134.25	63.01	50.68	34.25
G08	0.51	0.53	**–**3.92	0.45	11.76	0.29	43.14	0.22	56.86	103.92	88.24	56.86	43.14
G09	0.42	0.37	11.90	0.45	**–**7.14	0.25	40.48	0.18	57.14	88.10	107.14	59.52	42.86
G10	0.41	0.43	**–**4.88	0.36	12.20	0.23	43.90	0.16	60.98	104.88	87.80	56.10	39.02
G11	1.07	1.28	**–**19.63	1.32	**–**23.36	0.78	27.1	0.46	57.01	119.63	123.36	72.9	42.99
G14	0.28	0.25	10.71	0.29	**–**3.57	0.14	50.00	0.09	67.86	89.29	103.57	50.00	32.14
G18	0.36	0.26	27.78	0.37	**–**2.78	0.32	11.11	0.25	30.56	72.22	102.78	88.89	69.44
G26	0.14	0.12	14.29	0.17	**–**21.43	0.09	35.71	0.07	50.00	85.71	121.43	64.29	50.00
G28	0.26	0.28	**–**7.69	0.33	**–**26.92	0.18	30.77	0.10	61.54	107.69	126.92	69.23	38.46
G29	0.69	0.74	**–**7.25	0.77	**–**11.59	0.65	5.80	0.56	18.84	107.25	111.59	94.20	81.16
J01	0.63	0.75	**–**19.05	0.42	33.33	0.33	47.62	0.21	66.67	119.05	66.67	52.38	33.33
J04	0.43	0.46	**–**6.98	0.48	**–**11.63	0.35	18.6	0.25	41.86	106.98	111.63	81.4	58.14
J10	0.70	0.76	**–**8.57	0.82	**–**17.14	0.59	15.71	0.42	40.00	108.57	117.14	84.29	60.00
J11	1.12	1.13	**–**0.89	0.91	18.75	0.82	26.79	0.72	35.71	100.89	81.25	73.21	64.29
J24	0.38	0.42	**–**10.53	0.49	**–**28.95	0.35	7.89	0.14	63.16	110.53	128.95	92.11	36.84
J25	0.88	0.95	**–**7.95	0.93	**–**5.68	0.72	18.18	0.63	28.41	107.95	105.68	81.82	71.59
J62	1.06	1.09	**–**2.83	0.89	16.04	0.73	31.13	0.66	37.74	102.83	83.96	68.87	62.26
Y01	0.46	0.49	**–**6.52	0.52	**–**13.04	0.42	8.70	0.33	28.26	106.52	113.04	91.30	71.74
Y02	0.80	0.83	**–**3.75	0.87	**–**8.75	0.57	28.75	0.42	47.50	103.75	108.75	71.25	52.50
Y31	0.57	0.45	21.05	0.59	**–**3.51	0.52	8.77	0.37	35.09	78.95	103.51	91.23	64.91
Y45	0.35	0.36	**–**2.86	0.25	28.57	0.21	40.00	0.16	54.29	102.86	71.43	60.00	45.71
Y42	0.49	0.51	**–**4.08	0.42	14.29	0.33	32.65	0.27	44.90	104.08	85.71	67.35	55.10

The data in the table are the average of data.

The results of this study demonstrated that drought and water shortage have a significant effect on the proliferation of endophytic fungi. As drought stress increased, the proliferation of strains was partially inhibited. Table 3 shows that at a PEG-6000 concentration of 5%, the growth of strains G14, G18, G26, and Y31 was somewhat inhibited, while the proliferation of other strains remained unaffected. At a PEG-6000 concentration of 10%, the proliferation of strains G05, G06, G08, G10, J01, J11, J62, Y45, and Y42 was partially inhibited, while the growth of other strains was not affected. When the PEG-6000 concentration increased to 15 and 20%, the proliferation of all strains was partially inhibited, indicating that strain growth was suppressed with the intensification of drought stress.

Furthermore, it can be observed from the table that, while the proliferation of strains may be somewhat inhibited with increasing PEG-6000 concentration, the rate of decrease in OD value for the 25 strains (G14, G28, G11, G10, G08, G09, G01, J04, Y01, Y02, G02, G18, J24, Y45, G26, G06, Y42, G05, J10, G29, Y31, J01, J11, J25, and J62) is lower compared with other strains at the same PEG-6000 concentration. This suggests that these 25 strains exhibit a certain level of drought tolerance and could be further studied as potential drought-tolerant strains.

#### 3.2.2. Molecular identification of drought-tolerant endophytic fungi

To simulate drought stress, PEG-6000 was used in experiments conducted on endophytic fungi isolated from different tissues of *C. multiflorus*. A total of 25 drought-tolerant strains were screened. The ITS sequences of these 25 endophytic fungi were obtained through cloning sequencing. The obtained sequences were, then, compared with those available on NCBI (https://blast.ncbi.nlm.nih.gov/Blast.cgi?PROGRAM=blastn&PAGE_TYPE=BlastSearch&LINK_LOC=blasthome). The strain identification was based on the sequence with the highest similarity. Table 4 shows that the 25 drought-tolerant endophytic fungi belong to 7 genera and 12 species. These include *Aspergillus niger* (G14, G28, G11, G10, G08, G09, G01, J04, Y01, and Y02), *Aspergillus tubingensis* (G02, G18, J24, and Y45), *Aspergillus pseudosclerotiorum* (G26), *Aspergillus welwitschiae* (G06), *Aspergillus aculeatus* (Y42), *Fusarium oxysporum* (G05, J10), *Colletotrichum siamense* (G29), *Penicillium polonicum* (Y31), *Diaporthe vaccinii* (J01), *Geotrichum candidu* (J11), *Metarhizium anisopliae* (J25), and *Fusarium proliferatum* (J62). Among these, *Aspergillus* accounted for the majority, representing 14.17% of the total isolated strains. Specifically, 13 drought-tolerant strains were isolated from the root part, belonging to 3 genera and 6 species. From the stem part, 7 plants yielded strains belonging to 6 genera and 7 species. Partial separation from the leaves of 5 plants resulted in strains belonging to 2 genera and 4 species.

**Table 4:** Identification results of drought-tolerant endophytic fungi in *C. multiflorus*.

**Groups (genera)**	**Strain number**	**The closest strain (accession no.)**	**Sequence similarity/%**
*Aspergillus*	G14	*Aspergillus niger* (MT184804.1)	100
	G28	*Aspergillus niger* (ON208730.1)	99.82
	G11	*Aspergillus niger* (KX099623.1)	100
	G10	*Aspergillus niger* (MK543209.1)	100
	G08	*Aspergillus niger* (KX099623.1)	100
	G09	*Aspergillus niger* (MN792832.1)	100
	G01	*Aspergillus niger* (MK543209.1)	99.82
	J04	*Aspergillus niger* (MH855928.1)	100
	Y01	*Aspergillus niger* (MG991572.1)	100
	Y02	*Aspergillus niger* (KX099623.1)	100
	G02	*Aspergillus tubingensis* (KX664374.1)	99.91
	G18	*Aspergillus tubingensis* (KX664401.1)	100
	J24	*Aspergillus tubingensis* (KX664401.1)	100
	Y45	*Aspergillus tubingensis* (KX664371.1)	100
	G26	*Aspergillus pseudosclerotiorum* (OW985641.1)	99.47
	G06	*Aspergillus welwitschiae* (OL711714.1)	99.91
	Y42	*Aspergillus aculeatus* (MH892845.1)	100
*Fusarium*	G05	*Fusarium oxysporum* (MN749138.1)	100
	J10	*Fusarium oxysporum* (MN749138.1)	100
	J62	*Fusarium proliferatum* (OM956067.1)	99.80
*Colletotrichum*	G29	*Colletotrichum siamense* (MK184418.1)	99.62
*Penicillium*	Y31	*Penicillium polonicum* (MT487786.1)	99.81
*Diaporthe*	J01	*Diaporthe vaccinii* (AB470842.1)	99.63
*Geotrichum*	J11	*Geotrichum candidum* (KX218269.1)	98.77
*Metarhizium*	J25	*Metarhizium anisopliae* (MH859069.1)	99.61

According to the strain identification results and the drought-tolerant strain screening results, as shown in Table 3, 12 strains were selected for renaming in order to proceed with subsequent experiments. The renamed strains are as follows: G11 was named *Aspergillus niger* SXZ001; G02 was named *Aspergillus tubingensis* SXZ002; G26 was named *Aspergillus pseudosclerotiorum* SXZ003; G06 was named *Aspergillus welwitschiae* SXZ004; Y42 was designated *Aspergillus aculeatus* SXZ005; J10 was designated *Fusarium oxysporum* SXZ006; G29 was named *Colletotrichum siamense* SXZ007; Y31 named *Penicillium polonicum* SXZ008; J01 was named *Diaporthe vaccinia* SXZ009; J11 was designated *Geotrichum candidu* SXZ010; J25 was designated *Metarhizium anisopliae* SXZ011; and J62 was designated *Fusarium proliferatum* SXZ012.

### 3.3. Drought-tolerant endophytic fungi had potassium lysis, phosphorus dissolution, and IAA-producing activity screening results

#### 3.3.1. Phosphorus-soluble strain screening results

##### 3.3.1.1. Results of initial screening of phosphorus-lytic endophytic fungi plates

After preliminary screening, 12 endophytic fungi were cultured on inorganic phosphorus medium for 3 days, and seven of them were able to form a decomposition circle on inorganic phosphorus plates. These seven strains were *F. oxysporum* SXZ006, *A. welwitschiae* SXZ004, *A. niger* SXZ001, *A. tubingensis* SXZ002, *F. proliferatum* SXZ012, *P. polonicum* SXZ008, and *A. aculeatus* SXZ005 (Table 5). The ratio of decomposition circle diameter to colony diameter for these seven inorganic phosphorus-dissolving strains ranged from 1.21 to 1.60. Different strains exhibited different levels of phosphorus-dissolving activity. Among them, the phosphorus-solubilizing ability of strain *A. tubingensis* SXZ002 was higher than that of other strains, producing a phosphorus-soluble ring diameter of 1.60 cm, compared with strain *A. welwitschiae* SXZ004 and *P. Polonicum* SXZ008, which has poor phosphorus dissolving ability, and the diameter of the phosphorus ring is only 1.21 cm (Table 5).

**Table 5:** Preliminary screening results of phosphorus-soluble active strains.

**Strain number**	**Transparent circle D (cm)**	**Colony diameter d (cm)**	**Soluble index D/d (cm)**
*F. oxysporum* SXZ006	3.63 ± 0.14	2.89 ± 0.06	1.26 ± 0.07
*A. welwitschiae* SXZ004	3.33 ± 0.14	2.74 ± 0.13	1.21 ± 0.01
*A. niger* SXZ001	4.27 ± 0.18	3.23 ± 0.05	1.32 ± 0.04
*A. tubingensis* SXZ002	4.53 ± 0.53	2.83 ± 0.04	1.60 ± 0.15
*F. proliferatum* SXZ012	2.55 ± 0.07	1.65 ± 0.14	1.55 ± 0.12
*P. polonicum* SXZ008	2.40 ± 0.26	1.98 ± 0.15	1.21 ± 0.05
*A. aculeatus* SXZ005	4.70 ± 0.02	3.50 ± 0.08	1.34 ± 0.06

##### 3.3.1.2. Re-screening results of phosphate solubilizing strains in flasks

The phosphorus standard curve was plotted with the concentration of the phosphorus standard solution as the abscissa and the absorbance value of the solution at an OD value of 700 as the ordinate. The phosphorus content calculation formula is *y* = 1.2205^*^x+0.0072, the correlation coefficient *R*^2^ = 0.9979, and its correlation is greater than 0.9, and the soluble phosphorus content in the fermentation broth of the dissolved phosphorus strain can be calculated according to the corresponding absorbance value of the fermentation broth of the strain.

The seven strains with dissolved inorganic phosphorus activity obtained from the initial screening were inoculated into the inorganic phosphorus (NPA) liquid medium for flask shaking culture, and, the water-soluble phosphorus content in the fermentation broth was determined (Table 6). The results showed that the seven endophytic fungi had a certain solubility ability to inorganic phosphorus, but the phosphorus-soluble ability of different strains was different, and the soluble phosphorus content in the fermentation broth of each strain was between 101.98 and 414.51 μg^.^ml^−1^. Overall, the phosphorus dissolved content of the seven endophytic fungal strains was significantly higher than that of CK, and the water-soluble phosphorus content produced by the strains ranged from 91.50 to 404.03 μg^.^L^−1^ compared with CK, which exhibits obvious inorganic phosphorus decomposition ability. Among them, the soluble phosphorus content of *P. polonicum* SXZ008 fermentation broth was the highest, which was 414.51 μg^.^ml^−1^, which was significantly higher than that of other strains, followed by *A. niger* SXZ001, *F. oxysporum* SXZ006, and *A. aculeatus* SXZ005. There was no significant difference between the three strains, but they were significantly higher than other strains, indicating that their ability to dissolve inorganic phosphorus was basically the same and stronger than other strains. The strain with the least phosphorus solubilizing ability was *F. proliferatum* SXZ012 (101.98 μg^.^ml^−1^,) in comparison to *P. polonicum* SXZ008, which is significantly lower than that of other strains.

**Table 6:** Results of re-screening of phosphorus-soluble active strains.

**Strain number**	**Water-soluble phosphorus content (μg^.^mL^−1^)**
CK	10.48 ± 0.49f
*A. niger* SXZ001	302.35 ± 5.91b
*A. tubingensis* SXZ002	204.10 ± 25.97c
*F. oxysporum* SXZ006	282.25 ± 20.88b
*A. welwitschiae* SXZ004	127.61 ± 11.59d
*P. polonicum* SXZ008	414.51 ± 7.56a
*A. aculeatus* SXZ005	281.52 ± 9.14b
*F. proliferatum* SXZ012	101.98 ± 11.08e

The data in the table are mean ± standard deviation. Different lowercase letters of the same column data indicate that there are significant differences in phosphorus solubility of different strains (*P* < 0.05).

#### 3.3.2. Screening results of potassium-dissolving strains

##### 3.3.2.1. Results of initial screening of potassium-dissolving strains

Through the screening of potassium dissolving medium, 6 endophytic fungi with potassium dissolving properties were successfully screened from the above drought-tolerant strains, and a decomposition circle could be formed on potassium-dissolving medium, respectively *F. oxysporum* SXZ006, *A. welwitschiae* SXZ004, *A. niger* SXZ001, *P. polonicum* SXZ008, *A. aculeatus* SXZ005, and *A. tubingensis* SXZ002 (Table 7). The ratio of the diameter of the decomposition circle of potassium-lyzing active endophytic fungi to the diameter of the colony circle ranged from 1.05 to 1.35 cm. Among them, strain *A. niger* SXZ001 displayed strong potassium-dissolving activity, and the diameter of the potassium-dissolving circle formed is 1.35 cm, and strain *A. aculeatus* SXZ005 has poor potassium-dissolving ability, forming a potassium dissolving circle diameter of only 1.05 cm.

**Table 7:** Preliminary screening of potassium-lytic active strains.

**Strain number**	**Transparent ring D (cm)**	**Colony diameter d (cm)**	**Clear circle D/colony diameter d (cm)**
*F. oxysporum* SXZ006	4.13 ± 0.44	3.45 ± 0.31	1.20 ± 0.06
*A. welwitschiae* SXZ004	3.87 ± 0.01	3.31 ± 0.19	1.17 ± 0.06
*A. niger* SXZ001	3.87 ± 0.25	2.87 ± 0.21	1.35 ± 0.10
*P. polonicum* SXZ008	1.78 ± 0.17	1.55 ± 0.21	1.15 ± 0.05
*A. aculeatus* SXZ005	4.30 ± 0.06	4.10 ± 0.08	1.05 ± 0.05
*A. tubingensis* SXZ002	4.33 ± 0.26	3.28 ± 0.22	1.32 ± 0.03

##### 3.3.2.2. Results of re-screening of potassium-solubilizing strains

The six strains with potassium solubilization activity obtained from the initial screening were cultured with potassium solubilization liquid medium in a shaking flask, and the soluble potassium content in the fermentation broth was determined by atomic spectrophotometer (Table 8). The results showed that all the six endophytic fungi had certain potassium solubilizing activity, which was consistent with the initial screening results. However, there were some differences in potassium solubilizing ability among different strains. Overall, the potassium lysis content of the six endophytic fungal strains was significantly higher than that of CK, and the water-soluble potassium content produced by the strains ranged from 13.85 to 25.62 mg^.^l^−1^ compared with CK, showing obvious potassium feldspar powder decomposition ability. Among them, strain *A. niger* SXZ001 fermentation broth had the highest soluble potassium content (30.94 mg^.^l^−1^), followed by *A. tubingensis* SXZ002 (29.39 mg^.^l^−1^) and *F. oxysporum* SXZ006 (28.35 mg^.^l^−1^). The potassium lysis of these three endophytic fungi was significantly higher than that of other strains, indicating that the potassium dissolving ability of these three endophytic fungi was basically the same but stronger than that of other strains.

**Table 8:** The results of shaking flask re-screening of potassium-solubilizing strains.

**Strain number**	**k content (mg^.^L^−1^)**
CK	5.33 ± 0.04c
*A. niger* SXZ001	30.94 ± 2.05a
*A. tubingensis* SXZ002	29.39 ± 3.04a
*A. welwitschiae* SXZ004	21.76 ± 0.28b
*A. aculeatus* SXZ005	19.17 ± 1.00b
*F. oxysporum* SXZ006	28.35 ± 1.90a
*P. polonicum* SXZ008	20.46 ± 2.47b

The data in the table are mean ± standard deviation. Different lowercase letters of the same column data indicate that there are significant differences in potassium solubilization ability of different strains (*P* < 0.05).

#### 3.3.3. Results of IAA competency assessment

Drawing of IAA standard curve: taking the IAA solution concentration as the abscissa, the absorbance value at 530 nm was determined in the ultraviolet spectrophotometer as the ordinate, and the IAA standard curve was plotted. The standard curve of IAA was y = 0.0461 * x + 0.0003, with an *R*^2^ value of 0.9948. The correlation coefficient, which is 0.9948, is greater than 0.9, indicating that the IAA content in the strain's fermentation broth can be calculated according to the corresponding absorbance value of the strain's fermentation broth, and then determine whether the strain has the ability to produce IAA.

Through qualitative assay results, it was found that, compared with the control group, 6 of the 12 drought-tolerant strains had a certain ability to produce IAA, which were *A. pseudosclerotiorum* SXZ003, *F. oxysporum* SXZ006, *C. siamense* SXZ007, *D. vaccinii* SXZ009, *G. candidu* SXZ010, and *F. proliferatum* SXZ012. After adding the Salkowski colorimetric solution, the color depth of the fermentation broth was inconsistent, indicating that there was a certain difference in the ability of the strain to produce IAA. In addition, the IAA content of the supernatant of the strain fermentation broth was determined, and the results are shown in Table 9. According to the assay results, the yield of six strains of IAA ranged from 0.04 to 0.42 mg^.^ml^−1^. The content of IAA in the supernatant of *F. proliferatum* SXZ012 fermentation broth was 0.42 mg·ml^−1^, which was significantly higher than that of other strains, followed by *F. oxysporum* SXZ006 and *C. siamense* SXZ007, which were 0.16 mg·ml^−1^ and 0.20 mg·ml^−1^, respectively. The strains with the worst IAA production were *D. vaccini* SXZ009 and *G. candidu* SXZ010. The yield was only 0.04 mg·ml^−1^.

**Table 9:** IAA production of the strain.

**Strain number**	**IAA production mg^.^ml^−1^**
*D. vaccinii* SXZ009	0.04 ± 0.01c
*F. oxysporum* SXZ006	0.20 ± 0.07b
*G. candidu* SXZ010	0.04 ± 0.01c
*A. pseudosclerotiorum* SXZ003	0.06 ± 0.05c
*C. siamense* SXZ007	0.16 ± 0.05b
*F. proliferatum* SXZ012	0.42 ± 0.09a

The data in the table are mean ± standard deviation, and different lowercase letters in the same column indicate significant difference (*P* < 0.05).

In summary, among the 12 strains with drought tolerance screened from the endophytic fungi of *C. multiflorus*, there are 7 strains with phosphate solubilizing activity, among which the strains with strong phosphorus-soluble activity were *P. polonicum* SXZ008, *A. niger* SXZ001, *F. oxysporum* SXZ006, and *A. aculeatus* SXZ005. There were 6 strains with potassium-dissolving activity, among which the strains with strong potassium-dissolving activity were *F. proliferatum* SXZ012, *F. oxysporum* SXZ006, and *C. siamense* SXZ007. There are 6 strains capable of producing IAA, of which the strains with strong IAA production capacity are *F. proliferatum* SXZ012, *F. oxysporum* SXZ006, and *C. siamense* SXZ007.

## 4. Discussion

### 4.1. Isolation of culturable endophytic fungi from different parts of *C. multiflorus*

In this experiment, when using a PDA medium to isolate and purify endophytic fungi in different tissues of *C. multiflorus*, it was found that *C. multiflorus* is rich in endophytic fungal resources, but there are certain differences in the community composition of endophytic fungi in different tissues, and 113 endophytic fungi were isolated from different tissues of *C. multiflorus*, of which the stem part had the largest number of strains and fewest leaves, which was similar to the results by Jagannath et al. (2021) on endophytic fungi of *Baliospermum montanum* (Willd.) Muell. Arg, but there were differences with the results of most plant studies, such as with the study by Ahmad et al. (2022) The isolation results of endophytic fungi in different parts of *Acacia mangium* Willd. were different, with *Acacia mangium* having the most leaves, followed by stems and petioles and fewest fruits. Yanli et al. (2020) isolated the endophytic fungi of different tissues of the poisonous plant *Delphinium grandiflorum* L., which were the most common in flowers, followed by leaves, roots, and stems. In this study, it is believed that there is a lot of isolation of endophytic fungi in the stem, which may be because the stems of *C. multiflorus* have abundant nutrients, which are more suitable for the survival of endophytic fungi, or when the isolation of endophytic fungi is carried out, the root and leaf tissues are disinfected too much, resulting in the natural death or inactivation of some strains, similar to the research results by Zuowei et al. (2021). Few endophytic fungi were isolated from the leaves, which may be because the relative surface area of the leaves of *C. multiflorus* was small or the nutrients contained were less susceptible to strain infection than other parts, or the adaptability of endophytic fungi in the leaves was weak in artificial PDA medium, which was similar to the results by Shuhang et al. (2020). There are differences in endophytic fungi in different tissue distribution, which may be due to the different structures or chemical components of different organization, so the endophytic fungal communities that invade and grow in each organization are different. The reasons for the difference in the distribution of endophytic fungi need to be further studied, such as using different media to collect plants at different times and locations for isolation and culture of endophytic fungi to better reveal the distribution of endophytic fungi in *C. multiflorus* and tap the endophytic fungal resources of *C. multiflorus* (Huihua, 2015). To a certain extent, this study shows that different tissues of *C. multiflorus* are rich in endophytic fungal resources, which can provide a rich resource treasure trove for further screening of drought-tolerant and growth-promoting strains.

### 4.2. Screening and growth promotion evaluation of drought-tolerant fungi of *C. multiflorus*

The ITS sequence plays a crucial role in species identification and classification. Due to its high variability and specificity among species, the ITS sequence can be used to differentiate different species, genera, and families of endophytic fungi (O'Brien et al., 2005). In this experiment, ITS sequence identification was conducted on 25 drought-tolerant fungal strains, revealing that these strains belonged to 12 species from 7 genera. This, to some extent, demonstrates the high potential and importance of ITS sequencing in microbiological research, which can play a crucial role.

As one of the common abiotic stress factors in the process of plant growth and development, drought stress will have a great effect on plant morphology, physiology, and biomass accumulation and is a bottleneck that restricts the development of agriculture and forestry in many water-scarce areas. Therefore, studying crop drought stress and alleviating the harm of drought is of great significance to agricultural and forestry production and development (Deng et al., 2012). At present, most of the means used to solve plant drought stress are chemical regulation and molecular biology techniques. With the deepening of microbiological research, a large number of studies have proven that endophytic fungi also have important development values in improving the resistance of host plants to drought stress. Many researchers have isolated strains from plants that can significantly improve plant drought tolerance. For example, strains of *B. japonicum* 4788, *B. japonicum* 4792, and *B. japonicum* USDA110 screened by Xiaofeng (2012) have the ability to nodulate and fix nitrogen and promote plant growth under drought conditions. In this study, *Aspergillus* species such as *A. niger* SXZ001, *A. tubingensis* SXZ002, *A. welwitschiae* SXZ004, and *A. Echinospora* SXZ004 were also isolated *A. aculeatus* SXZ005, *F. oxysporum* SXZ006, and *F. proliferatum* SXZ012 of *Fusarium* showed good tolerance to drought stress. The relevant literature reported that most strains of these two genera have strong osmotic pressure tolerance (Araújo et al., 2020), such as Ullah et al. (2021) found that *Aspergillus* has a high abundance in cotton rhizosphere soil under drought stress. Beever and Laracy (1986) found that *Aspergillus nidulans* has drought tolerance. Xiaojing (2019) found that *Fusarium* endophytic fungi isolated from *Elymus dahuricus* Turcz. can improve the drought tolerance of wheat by improving osmoregulation, antioxidant enzyme activity, and photosynthetic properties under drought stress and promoting plant uptake of carbon, nitrogen, and zinc. The results of this study are consistent with these findings. In addition, these two genera also have a wide range of uses, *Aspergillus* is a class of filamentous fungi, with wide varieties, rich in metabolites, and have a wide range of application value in agriculture, medicine, bioenergy, cosmetics, food fermentation, and other industries (Frisvad and Larsen, 2015; Parent-Michaud et al., 2020; Yu et al., 2020).

*Fusarium*, as an important plant pathogen, can also play an important role in agricultural and forestry production as a biocontrol pathogen. In addition to drought tolerance and wide use, *Aspergillus* and *Fusarium* fungi are also important phosphorus-solubilizing and potassium-dissolving microorganisms, capable of secreting cellulase, acting on the decomposition of carbon, and participating in the dissolution of poorly soluble phosphorus in soil, which can improve the ability of plants to absorb and utilize soluble phosphorus and potassium in soil (Vassilev et al., 1995; Narsian and Patel, 2000; Xiaorong and Qimei, 2001; Meena et al., 2017). In this study, *A. niger* SXZ001, *A. tubingensis* SXZ002, *A. welwitschiae* SXZ004, *A. aculeatus* SXZ005, *F. oxysporum* SXZ006, and *F. proliferatum* SXZ012, which were screened by Ca_3_ (PO_4_)_2_ and Potassium feldspar powder, had significant ability to dissolve phosphorus and potassium. Compared with the control group, the soluble phosphorus and the potassium contents were significantly increased. There have been many studies on the phosphorus-dissolving ability of *Penicillium* strains at home and abroad, including *P. oxalicum, P. aculeatum*, and *P. bilaiae*, among which *P. bilaiae* is widely used to increase crop yield (Aikaterini et al., 2018; Gómez-Muñoz et al., 2018). In this study, the phosphate-solubilizing ability of *P. polonicum* SXZ008 endophytic fungi isolated from *C. multiflorus* was significantly higher than that of other strains. In addition, the ability of *A. welwitschiae* SXZ004 isolated from *C. multiflorus* to dissolve phosphorus and potassium has not been reported, and its specific mechanism of promoting plant growth needs further study.

IAA, as a class of endogenous auxins that can increase plant growth and promote plant root formation, plays a very important role in the process of plant growth and development (Kashyap et al., 2023). It can be found not only in plants but also in fungi (Spaepen and Vanderleyden, 2011), such as Huong et al. (2022), who found that from stevia [*Stevia rebaudiana* (Bertoni) Bertoni] endophytic fungi, *Penicillium simplicissimum* CN7 and *Talaromyces flavus* BC1 separated from the root part and *Trichoderma konilangbra* DL3 have the ability to produce IAA. When, Chen et al. (2011) studied the endophytic fungus of *Rehmannia glutinosa* (Gaert.) Libosch. ex Fisch. et Mey., they found that the isolated endophytic fungus IV. of the genus *Ceratobasidium* had the ability to produce IAA, and the IAA content could reach 5.712 mg^.^l^−1^. In this study, six drought-tolerant strains had the ability to produce IAA, and the content of IAA was between 0.04 and 0.42 mg·l^−1^. These strains belonged to *Aschersonia, Fusarium, Geotrichum, Aspergillus*, and *Colletotrichum*, respectively. Among them, the number of IAA-producing strains isolated from *Fusarium* was more, which proved that the *Fusarium* endophytic fungi in *C. multiflorus* were potential beneficial fungi, and their ability to produce IAA was similar to that of endophytic fungi isolated from other plants (Hoffman et al., 2017; Shi et al., 2017).

The results showed that there were abundant endophytic fungal strains in different tissues of *C. multiflorus*. Moreover, some of these strains showed functional characteristics of drought tolerance and growth promotion. To a certain extent, this study clarified that some endophytic fungi of *C. multiflorus* have drought tolerance and growth-promoting functions, which can provide a new variety of resources for plant drought tolerance and growth-promoting fungi, and provide a theoretical basis for the potential application of endophytic fungi of *C. multiflorus* in improving plant tolerance in agricultural and forestry production.

## Data availability statement

The datasets presented in this study can be found in online repositories. The names of the repository/repositories and accession number(s) can be found in the article/Supplementary material.

## Author contributions

Z-WL: Conceptualization, Formal analysis, Investigation, Methodology, Writing—original draft, Writing—review & editing. H-YL: Conceptualization, Formal analysis, Investigation, Methodology, Writing—original draft. C-LW: Data curation, Investigation, Software, Writing—original draft. XC: Investigation, Resources, Software, Writing—original draft. Y-XH: Investigation, Methodology, Writing—original draft. M-MZ: Methodology, Software, Writing—original draft. Q-LH: Formal analysis, Methodology, Writing—original draft. G-FZ: Data curation, Funding acquisition, Project administration, Supervision, Validation, Writing—review & editing.
