# Supplementary material for: Isolation of endophytic fungi from Cotoneaster multiflorus and screening of drought-tolerant fungi and evaluation of their growth-promoting effects
Source: Front Microbiol. 2023 Nov 2;14:1267404. doi: 10.3389/fmicb.2023.1267404 (PMC10653309; doi:10.3389/fmicb.2023.1267404)

1、Phosphorus standard curve.





2、IAA standard solution curve.





3、Phylogenetic tree constructed by ITS sequences of drought-tolerant endophytic fungi isolated from different tissues of *C. multiflorus*


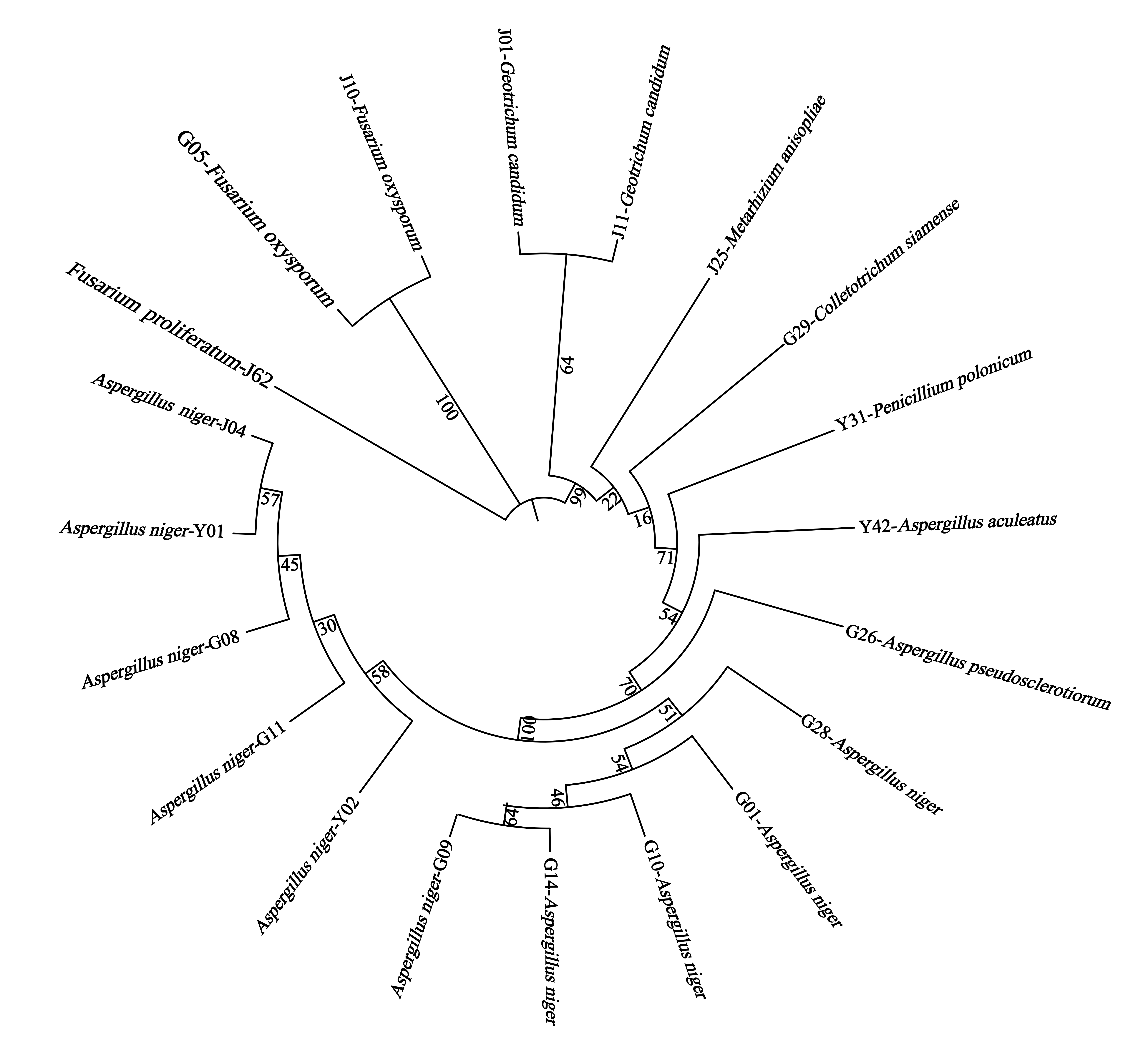

Supplement: Supplementary file 1 [file Table_1.docx]
